# Metabolic Effects of the Sweet Protein MNEI as a Sweetener in Drinking Water. A Pilot Study of a High Fat Dietary Regimen in a Rodent Model

**DOI:** 10.3390/nu11112643

**Published:** 2019-11-04

**Authors:** Rosa Cancelliere, Serena Leone, Cristina Gatto, Arianna Mazzoli, Carmine Ercole, Susanna Iossa, Giovanna Liverini, Delia Picone, Raffaella Crescenzo

**Affiliations:** 1 Department of Biology, Federico II University, Via Cintia, 80126 Naples, Italy; cancelliererosa@gmail.com (R.C.); cristina.gatto@unina.it (C.G.); arianna.mazzoli@unina.it (A.M.); susanna.iossa@unina.it (S.I.); liverini@unina.it (G.L.); 2 Department of Chemical Sciences, Federico II University, Via Cintia, 80126 Naples, Italy; serena.leone@unina.it (S.L.); ercoleca@hotmail.com (C.E.)

**Keywords:** sweet protein, MNEI, fructose, high fat diet

## Abstract

Sweeteners have become integrating components of the typical western diet, in response to the spreading of sugar-related pathologies (diabetes, obesity and metabolic syndrome) that have stemmed from the adoption of unbalanced dietary habits. Sweet proteins are a relatively unstudied class of sweet compounds that could serve as innovative sweeteners, but their introduction on the food market has been delayed by some factors, among which is the lack of thorough metabolic and toxicological studies. We have tried to shed light on the potential of a sweet protein, MNEI, as a fructose substitute in beverages in a typical western diet, by studying the metabolic consequences of its consumption on a Wistar rat model of high fat diet-induced obesity. In particular, we investigated the lipid profile, insulin sensitivity and other indicators of metabolic syndrome. We also evaluated systemic inflammation and potential colon damage. MNEI consumption rescued the metabolic derangement elicited by the intake of fructose, namely insulin resistance, altered plasma lipid profile, colon inflammation and translocation of lipopolysaccharides from the gut lumen into the circulatory system. We concluded that MNEI could represent a valid alternative to fructose, particularly when concomitant metabolic disorders such as diabetes and/or glucose intolerance are present.

## 1. Introduction

The major changes in dietary habits observed over the past decades, with the ready availability of sugars and processed food, have led, both in wealthy and developing countries, to an increase in metabolism-related diseases. In response to this trend, natural and artificial sweeteners have been massively introduced in the food and beverage market as sugar substitutes. While able to reduce the calorie intake from sweetened food, these compounds are often flawed, for instance by the occurrence of a bitter aftertaste, the unsuitability for certain preparations and, in some cases, repercussions on human health [1]. Recently, harmful effects of the consumption of artificial sweeteners on the gut microflora, with consequences on glucose tolerance, have emerged [2]. Even sweeteners such as fructose are not devoid of risks: in fact, we have demonstrated that a fructose-rich diet induces metabolic syndrome, inflammation and oxidative stress in a rat model of obesity [3,4]. A relatively unexplored class of sweet compounds is constituted by Sweet Proteins (SPs), which were first isolated in the 1970s from the berries and seeds of a few unrelated tropical plants [5,6]. Due to a sweetening power up to five orders of magnitude greater than sucrose [6], they are attractive starting points for the design of a next generation of sweeteners. The cellular utilization of 1 g of SPs, after digestion and absorption, similar to any other protein, produces 4 calories, however, since only small amounts need to be added to food to achieve intense sweetness, they provide almost no calorie contribution. Unlike most artificial sweeteners, they are also characterized by a taste quality that in some cases is comparable to that of sucrose [7]. The use of SPs has been limited by two main elements: first, their poor resistance to physical factors, such as temperature or pressure, which can affect the protein fold and, consequently, taste. Fortunately, this problem can be solved by molecular engineering, through the introduction of amino acid mutations enhancing resistance or taste [8,9,10,11]. Second, despite a long history of use among indigenous populations, extensive toxicological and physiological data are still lacking. Thus far, only one sweet protein, thaumatin, has been deemed safe and authorized by regulatory agencies for consumption and sale in the European Union, as it does not exhibit acute or chronic toxicity in rodents [12]. Preliminary genotoxicity, mutagenicity and ecotoxicity studies on MNEI provided positive results [13]; however, aside from toxicity, the metabolic consequences of the consumption of SPs are almost completely unexplored. These molecules seem very attractive for people with pathologies related to carbohydrate metabolism (i.e., diabetes, hyperlipemia, obesity), who need to remove sucrose and fructose from their diet and are often forced to adopt restricted regimens. Thus, studies on the effects of SPs in terms of insulin sensitivity, body weight increase and/or markers of inflammation are much needed to define their true potential. 

Here, we focused on MNEI, a single chain derivative of the plant sweet protein monellin and a model SP [14]. We used a rodent model to evaluate the main impact of the consumption of this SP and to assess its metabolic effects in terms of insulin sensitivity and body weight gain. Our studies have been performed on a well-defined animal model for human obesity, i.e., adult sedentary rats chronically fed a diet rich in fat and fructose, which reproduces the typical “western lifestyle”, characterized by the consumption of energy-dense, lipid-rich foods and fructose-rich beverages [15]. This allowed us to test the effects of the substitution of fructose with SPs in beverages. We assessed the degree of obesity, whole body composition, energy balance, plasma lipid profile and insulin sensitivity. Skeletal muscle lipid profile (triglycerides and ceramides) and insulin signaling (Akt and Erk phosphorylation) have been also estimated. Finally, we measured plasma levels of lipopolysaccharides (LPS) and tumor necrosis factor α (TNF-α) as markers of potential systemic inflammation and plasma activities of alanine aminotransferase (ALAT) and aspartate aminotransferase (ASAT) as general markers of tissue injury. Colon damage was estimated by measuring myeloperoxidase (MPO) activity and TNF-α levels in this tissue. As very little is known on the effects produced by sweet proteins in terms of nutritional profile, these data contribute to expanding our knowledge on sweet proteins and their potential use as sugar substitutes.

## 2. Materials and Methods

### 2.1. General Study Design

All experimental procedures involving animals were approved by “Comitato Etico-Scientifico per la Sperimentazione Animale” of the University of Naples Federico II (authorization n 2012/0024688). This work complies with the animal ethics principles and regulations of the Italian Health Ministry.

Male Wistar rats (Charles River, Calco, Italy) were adapted to room and cage environments for at least one week prior to the start of the experiment. In particular, 28 animals were caged singly in a temperature-controlled room (23 °C ± 1 °C) with a 12-h light/dark cycle. After this period of adaptation, seven rats (about 13 weeks-old) were euthanized for determination of initial body composition, while another seven rats were fed a high fat (lard-rich) diet for 30 days and drinking water was sweetened with 0.004% MNEI (HFM group, *n* = 7). Negative and positive controls were represented, respectively, by a group of seven rats fed low fat diet (LF group, *n* = 7) and by a group of seven rats fed the lard-rich diet, with drinking water sweetened with 10% fructose (HFF group, *n* = 7). This last diet was used to simulate the typical human “western lifestyle”. The 10% fructose and 0.004% MNEI solutions are iso-sweet [7]; therefore, the sweet solution utilized here would mimic a hypothetic MNEI-sweetened beverage for human consumption. The composition of the diets is detailed in Table 1. Recombinant protein was produced in *Escherichia coli* BL21(DE3) cells harboring the pET-22b+ plasmid containing the gene coding for MNEI and purified from the cell lysate by a coupled anion/cation exchange procedure, as previously described [16]. Sample purity and folding were confirmed by sodium dodecyl sulphate-polyacrylamide gel electrophoresis (SDS-PAGE), size exclusion chromatography and circular dichroism analysis. At the start of the experimental period, seven rats were euthanized for measurements of initial body energy and lipid content. During the treatment period of 30 days, body weight and food intake (adjusted for spillage) were monitored daily, while the feces, produced in the entire period, were collected to determine metabolizable energy (ME) intake. At the end of the experimental period, glucose tolerance tests were performed on the three groups of rats, and the animals were euthanized on the following day. Blood, epidydimal fat, hindleg skeletal muscle and colon were collected, while the carcasses were used for body composition determination.

### 2.2. Determination of Body Composition and Energy Balance

Guts were cleaned of undigested food and the carcasses were then autoclaved. After homogenization of the carcasses with a Polytron homogenizer (Kinematica, Lucerne, Switzerland), the resulting homogenates were frozen at −20 °C until the day of measurements. Duplicate samples of the homogenized carcass were analyzed for energy content by bomb calorimeter. Total body fat content was measured by the Folch extraction method [17]. Total body water content was determined by drying carcass samples in an oven at 60 °C for 48 h. Energy balance measurements were conducted by the comparative carcass technique as detailed previously [18]. Energy expenditure was determined as the difference between energy gain and ME intake, while energetic efficiency was calculated as the percentage of body energy retained per ME intake. 

### 2.3. Glucose Tolerance Test and Plasma Parameters

After 6 h of fasting, a first sample was obtained from venous blood from a small tail clip to determine basal glycemia and insulinemia, which were used for the determination of homeostasis model assessment (HOMA index) [19]. Following a glucose load (2g/kgbw, i.p. injection), the blood was collected after 20, 40, 60, 90, 120 and 150 min. The blood samples were centrifuged at 1400 × g_av_ for 8 min at 4 °C and plasma was stored at −20 °C. Plasma glucose concentration was obtained by colorimetric enzymatic method (Pokler Italia, Italy). Plasma insulin concentration was measured using an ELISA kit (Diametra, Spello, Italy) in a single assay to remove inter-assay variations. 

TNF-α concentration in plasma was determined using a rat specific ELISA (R&D Systems, MN, USA) according to manufacturer’s instruction. Briefly, the wells of a microtiter plate were coated with 100 µL of mouse anti-rat TNF-α (4 µg/mL) in PBS (137 mM NaCl, 2.7 mM KCl, 8.1 mM Na_2_HPO_4_, 1.5 mM KH_2_PO_4_, pH 7.4), and incubated overnight at room temperature. The antibody excess was then removed by washing with buffer (containing 0.05% (*v/v*) Tween 20 in PBS, pH 7.4), and the remaining sites on the plate were blocked with reagent diluent (PBS containing 1% BSA) (1 h, room temperature). After extensive washing with buffer solution, samples were added to the wells and incubated for 2 h at room temperature. After further washing with buffer solution, the wells were incubated with biotinylated goat anti-rat TNF-α (225 ng/mL in reagent diluent) followed by treatment with Streptavidin-HRP (1:200 dilution; 1h, room temperature). Peroxidase-catalyzed color development from o-Phenylenediamine was measured at 492 nm.

Plasma LPS determinations were performed by a Limulus amoebocyte lysate (LAL) kit (Lonza, Basel, Switzerland) using a LAL extract according to the manufacturer’s instruction. Briefly, samples were mixed with the LAL reagent and chromogenic substrate reagent for 16 min and absorbance readings were performed on a plate reader at 405 nm. 

Plasma triglycerides, cholesterol, ALAT and ASAT were measured by colorimetric enzymatic method using commercial kits (SGM Italia, Italy).

### 2.4. Composition, Inflammation and Insulin Signaling in Skeletal Muscle

Skeletal muscle lipids were determined by the same extraction method used for carcass [17], while skeletal muscle triglycerides were measured by the same colorimetric enzymatic kit used for plasma.

Skeletal muscle ceramide content was evaluated by ELISA as reported previously [20]. In brief, muscle lipids extracted with the Folch method (70 μL in methanol) were adsorbed to well bottoms overnight at 4 °C. Plates were blocked with 10 mM PBS, 140 mM NaCl, 0.1% Tween, pH 7.4, supplemented with 1% BSA for 1 h at 37 °C. The plates were then washed three times with 10mM PBS, 140mM NaCl, 0.05% Tween, pH 7.4 (Tween-PBS), and incubated with monoclonal anti-ceramide antibody (2 μg/mL) for 1 h at 37 °C. After three washes in Tween-PBS, peroxidase-conjugated goat anti-mouse immunoglobulin M (1:5000 dilution) was incubated for 1 h at 37 °C. After four washes in Tween-PBS, the wells were incubated with 100 μL of a color development solution (20 mg of o-phenylenediamine dihydrochloride in 50 mL of 70 mM Na_2_HPO_4_, 30 mM citric acid, pH 5, supplemented with 120 μL of 3% H_2_O_2_). After 15 min at 37 °C, the reaction was stopped by the addition of 50 μL of 2.5 M H_2_SO_4_ and the absorbance was measured at 492 nm. All tests were carried out in triplicate. Immunoreactivity was normalized to the starting tissue weight. Negative control reactions omitted the primary antibody.

TNF-α concentration in protein extracts from skeletal muscle was determined using a rat-specific ELISA (R&D Systems, MN, USA), as described for plasma samples.

For western blot determinations, skeletal muscle samples were homogenized in lysis buffer containing 20 mM Tris-HCl (pH 7.5), 150 mM NaCl, 2.7 mM KCl, 5% (*v/v*) glycerol, 1% (*v/v*) Triton X-100 and 50 µL/g tissue of protease inhibitor cocktail (all from Sigma-Aldrich, MO, USA) using a Potter homogenizer, shaken for 10 min at room temperature, and centrifuged at 14,000 × g_av_ for 30 min at 4 °C. The supernatants were collected and aliquots were denatured in a buffer (60.0 mM Tris pH 6.8, 10% sucrose, 2% SDS, 4% β-mercaptoethanol) and loaded onto a 12% SDS-Polyacrylamide gel. After the run in electrode buffer (50 mM Tris, pH 8.3, 384 mM glycine, 0.1% SDS), the gels were transferred onto polyvinylidene difluoride membranes (Immobilon-P, Merck Millipore, Germany) at 0.8 mA/cm2 for 90 min. The membranes were preblocked in blocking buffer (PBS; 3% BSA; 0.3% Tween 20) for 1 h and then incubated overnight at 4 °C with a primary antibody for pAkt (Cell Signaling, MA, USA, diluted 1:1000 in blocking buffer), pErk (Cell Signaling, MA, USA, diluted 1:1000 in blocking buffer) and serine palmitoyl transferase (SPT) (Abcam, Cambridge, UK, diluted 1:1000 in blocking buffer). The membranes were washed and then incubated for 1 h at room temperature with an HRP-conjugated secondary antibody (Promega, WI, USA). The membranes were finally washed, rinsed in distilled water and incubated at room temperature with a chemiluminescent HRP substrate (Immobilon, Millipore Corporation, MA, USA). Quantitative densitometry of the bands was carried out by Image Lab Software (Bio-Rad Laboratories, CA, USA). Akt was detected with polyclonal antibody (Cell Signaling, MA, USA, diluted 1:1000 in blocking buffer) and used to normalize the pAkt signal, Erk was detected with monoclonal antibody (Cell Signaling, MA, USA, diluted 1:1000 in blocking buffer) and used to normalize pErk signal, NFkB was detected with monoclonal antibody (Santa Cruz Biotechnology, TX, USA, diluted 1:1000 in blocking buffer) and used to normalize pNFkB (1:500 in blocking buffer) signal, while actin was detected with polyclonal antibody (Sigma-Aldrich, MO, USA; diluted 1:1000 in blocking buffer) and used to normalize SPT signal.

### 2.5. Markers of Inflammation and Leakiness in the Colon

The determination of MPO activity can be used as a surrogate marker of inflammation, since it has been shown that the activity of MPO solubilized from the inflamed tissue is directly proportional to the number of neutrophils seen in histologic sections [21]. MPO activity was therefore assessed in colon samples as reported by Kim et al. [22]. Briefly, tissue samples (100 mg) were homogenized in 1 mL of hexadecyltrimethylammonium bromide (HTAB) buffer (0.5% HTAB in 50 mM phosphate buffer, pH 6.0) and centrifuged at 13,400 × g_av_ for 6 min at 4 °C. MPO activity was measured spectrophotometrically: 10 µL of supernatant were combined with 200 µL of 50 mM phosphate buffer, pH 6.0, containing 0.167 mg/mL 0-dianisidine hydrochloride and 1.25% hydrogen peroxide. The change in absorbance at 450 nm was measured and one unit of MPO activity was defined as that degrading 1 µmol of peroxide per min at 25 °C.

TNF-α concentration in protein extracts from colon was determined using a rat specific ELISA (R&D Systems, MN, USA), as described for plasma samples.

Western blot of zonula occludens-1 (ZO-1), a tight junction protein, was performed on colon extracts with the same method described for skeletal muscle proteins, by using the ZO-1 polyclonal antibody (Thermo Fisher Scientific, IL, USA, diluted 1:500 in blocking buffer), while actin (Sigma-Aldrich, MO, USA, diluted 1:5000 in blocking buffer) was used for normalization of ZO-1 signal. The membranes were incubated with an AP-conjugated secondary antibody (Thermo Fisher Scientific, IL, USA) and then with a chemiluminescent AP substrate (CDP Star, Sigma-Aldrich, MO, USA). Chemiluminescent signals were detected by exposing autoradiography films (Eastman Kodak Company, NY, USA) to the membranes and quantitative densitometry of the bands was conducted as previously described.

### 2.6. Data Analysis and Statistics

Data are given as means ± SEM. Statistical analyses were performed by one-way ANOVA followed by Tukey post-test. Probability values less than 0.05 were considered to indicate a significant difference. All analyses were performed using GraphPad Prism 4 (GraphPad Software, CA, USA).

### 2.7. Chemicals

All of the chemicals employed in this research were of analytical grade and purchased from Sigma-Aldrich (MO, USA)

## 3. Results

Through a critical overview of the results obtained in this study, we observed that the variability of responses among rats within each group was very low, whatever the parameter analyzed.

### 3.1. Whole Body Compartments

Through the analysis of data relative to animal treatment, we observed that HFM rats had a similar water intake compared to control LF group (Table 2). The evaluation of body composition revealed a similar body weight gain in the three groups of rats and comparable amounts of total body lipids and epididymal fat between high fat fed rats (Table 2). Additionally, the analysis of parameters of energy balance showed the absence of significant variations between HFF and HFM groups, since metabolizable energy intake, energy expenditure, energy gain and metabolic efficiency were unchanged. 

### 3.2. Plasma Markers of Inflammation and Lipid Profile

**Figure 1:**
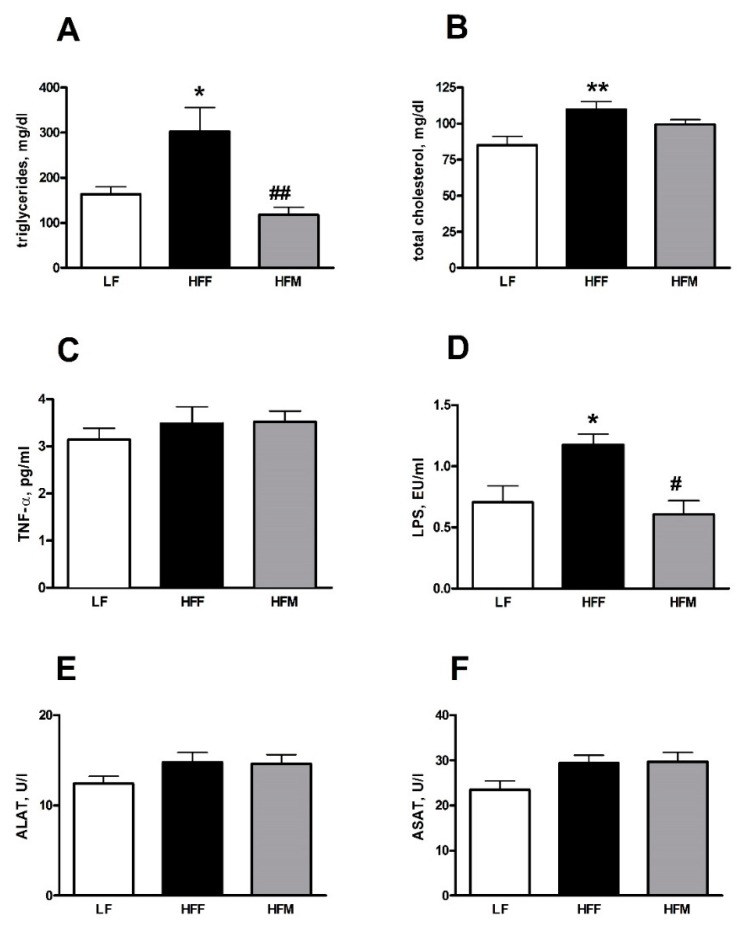
Plasma triglycerides (**A**), total cholesterol (**B**), TNF-α (**C**), LPS (**D**) levels, ASAT (**E**) and ALAT (**F**) activities in rats fed a low fat (LF), high fat plus fructose (HFF) or high fat plus MNEI (HFM) diet for 30 days. Figure shows means ± SEM for seven rodents in each group: LF, HFF and HFM. * *p* < 0.05 compared to LF, ** *p* < 0.01 compared to LF, ^#^ *p* < 0.05 compared to HFF, ^##^ *p* < 0.01 compared to HFF (one-way ANOVA followed by Tukey post-test). TNF-α: tumor necrosis factor-α; LPS: lipopolysaccharides; ALAT: alanine aminotransferase; ASAT: aspartate aminotransferase.

Figure 1 illustrates the impact of treatments on plasma markers of inflammation and liver injury and also shows the lipid profiles, which evidenced a clear negative effect of HFF. In fact, we found a significant increase in plasma triglycerides in HFF rats compared to LF group, while this parameter significantly decreased in HFM rats compared to HFF group (Figure 1A). Total plasma cholesterol significantly increased only in HFF rats compared to LF controls (Figure 1B). Plasma TNF-α levels were not affected by dietary treatment (Figure 1C), while LPS levels increased significantly in HFF rats, compared to LF controls, and decreased in HFM rats compared to HFF group (Figure 1D). Measurements of plasma ALAT and ASAT activities, which are markers of potential damage to the liver, show no significant variations between the three groups (Figure 1E,F).

### 3.3. Glucose Tolerance and Skeletal Muscle Lipid Profile

**Figure 2:**
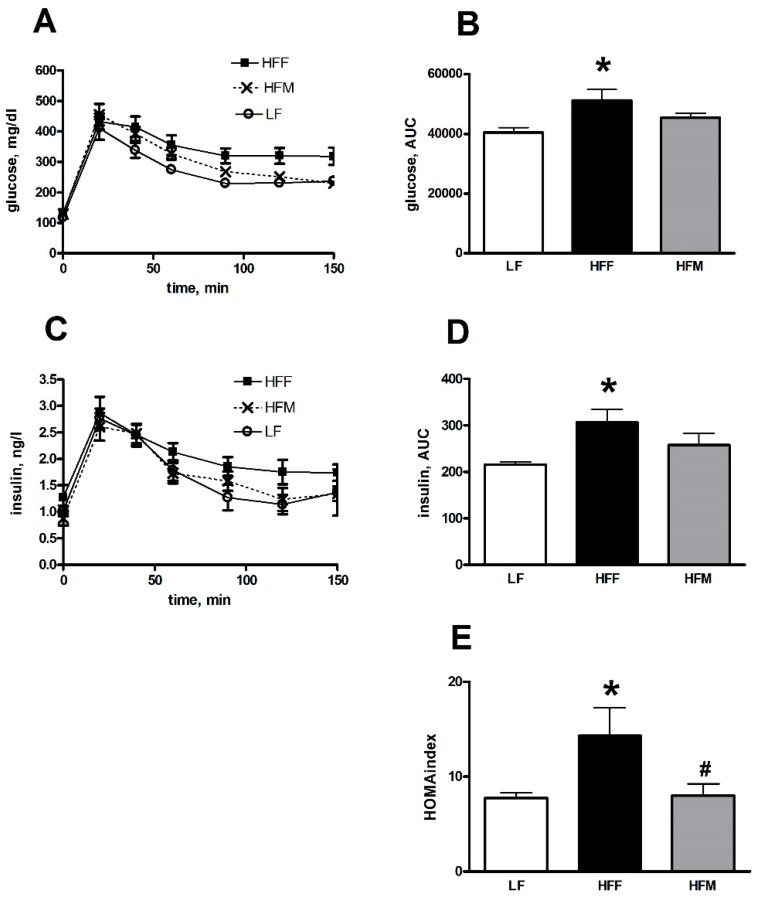
Glycemia (**A**), insulinemia (**C**) and the corresponding areas under the curve (AUC) (**B**,**D**) during intraperitoneal glucose tolerance test and HOMA index (**E**) in rats at the end of 30 days treatment with a low fat (LF), high fat plus fructose (HFF) or high fat plus MNEI (HFM) diet. Figure shows means ± SEM for seven rodents in each group: LF, HFF, HFM. * *p* < 0.05 compared to LF (one-way ANOVA followed by Tukey post-test). HOMA: homeostasis model assessment.

Glucose tolerance tests were performed at the end of dietary treatment (Figure 2). The HFF rats exhibited higher levels of both plasma glucose (Figure 2A) and insulin (Figure 2C) after the intraperitoneal glucose load compared to LF controls, as evidenced by the estimation of corresponding areas under the curves (Figure 2B,D). The calculation of HOMA confirmed these data, since this index of hepatic insulin resistance significantly increased in HFF rats compared to controls LF and was rescued in HFM group.

**Figure 3:**
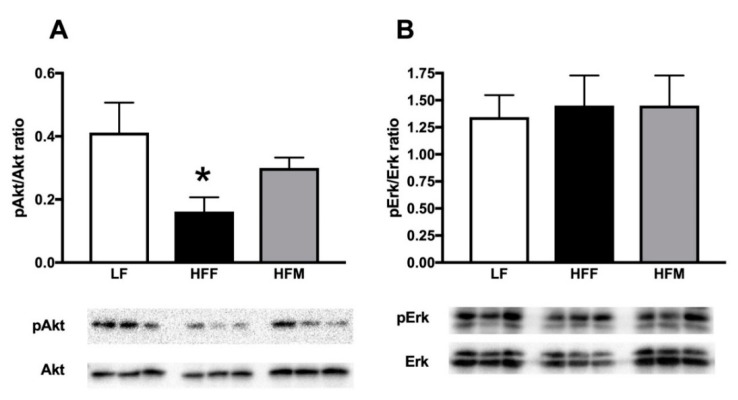
pAkt (**A**) and pErk (**B**) levels with representative blots in skeletal muscle from rats fed a low fat (LF), high fat plus fructose (HFF) or high fat plus MNEI (HFM) diet for 30 days. Figure shows means ± SEM for seven rodents in each group: LF, HFF and HFM. * *p* < 0.05 compared to LF (one-way ANOVA followed by Tukey post-test).

The activation of the insulin signaling pathway in skeletal muscle (Figure 3), via phosphorylation of Akt protein, was significantly reduced in HFF rats compared to LF controls, while sensitivity to insulin signaling was restored in HFM rats (Figure 3A). No variation was found in the expression of phosphorylated Erk protein (Figure 3B).

**Figure 4:**
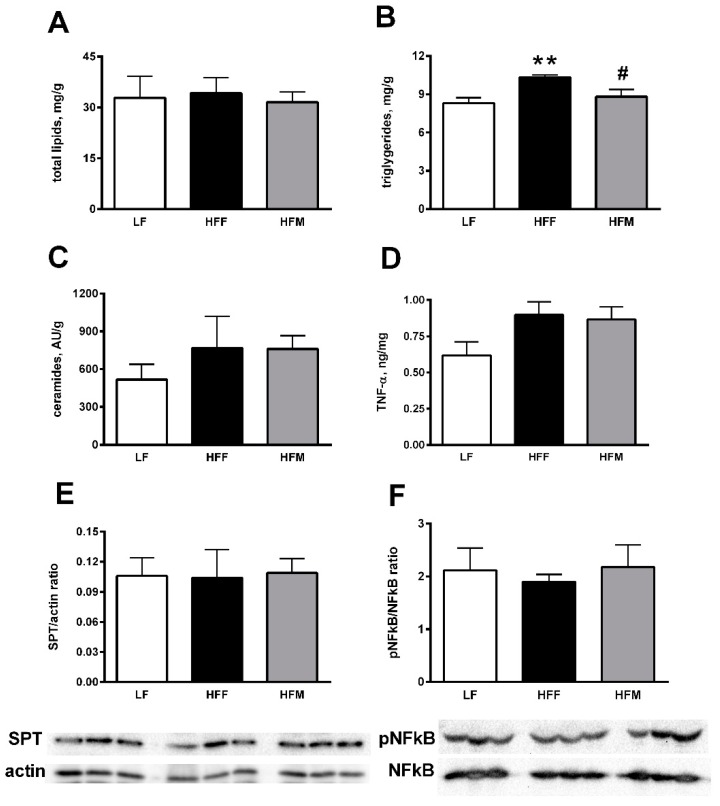
Total lipids (**A**), triglycerides (**B**), ceramides (**C**) and TNF-α (**D**), as well as SPT and pNFkB levels with representative blots (**E**,**F**) in skeletal muscle from rats fed a low fat (LF), high fat plus fructose (HFF) or high fat plus MNEI (HFM) diet for 30 days. Figure shows means ± SEM for seven rodents in each group: LF, HFF and HFM. ** *p* < 0.01 compared LF, ^#^ *p* < 0.05 compared to HFF (one-way ANOVA followed by Tukey post-test). SPT: serine palmitoyl transferase.

Skeletal muscle lipid profile as well as markers of inflammation are shown in Figure 4. Tissue triglycerides significantly increased in HFF rats compared to LF controls, and significantly decreased in HFM group, compared to HFF rats (Figure 4B). The levels of total lipids (Figure 4A), as well as ceramides (Figure 4C) and SPT content (Figure 4E), were unaffected by the diets here used. Levels of TNF-α in skeletal muscle were unchanged between 3 groups of rats (Figure 4D), as well as the phosphorylation of NFkB protein (Figure 4F).

### 3.4. Colon Markers of Inflammation and Leakiness

**Figure 5:**
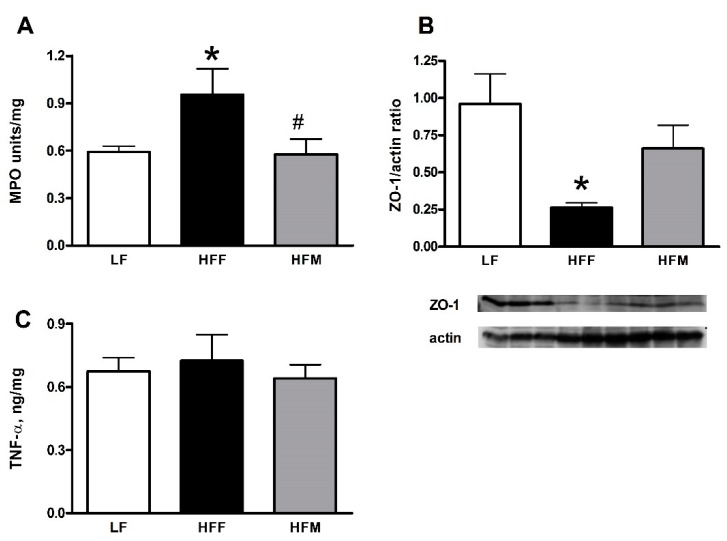
MPO activity (**A**), ZO-1 (**B**) and TNF-α (**C**) content in colon from rats fed a low fat (LF), high fat plus fructose (HFF) or high fat plus MNEI (HFM) diet for 30 days. Figure shows means ± SEM for seven rodents in each group: LF, HFF and HFM. * *p* < 0.05 compared to LF, ^#^ *p* < 0.05 compared to HFF (one-way ANOVA followed by Tukey post-test). MPO: myeloperoxidase; ZO-1: zonula occludens-1; TNF-α: tumor necrosis factor-α.

Markers of inflammation and leakiness in the colon are reported in Figure 5. MPO activity in this tissue increased in HFF rats compared to LF controls and decreased in HFM compared to HFF rats (Figure 5A). TNF-α levels were not affected by dietary treatment (Figure 5C). Finally, the content of ZO-1, a marker of leakiness in the colon, decreased in HFF rats compared to LF controls, while this parameter was rescued in HFM rats (Figure 5B).

## 4. Discussion

This study explored the metabolic effects of substituting the non-nutritive sweetener MNEI, a sweet protein, for the natural caloric sweetener fructose in beverages, in a regimen of high fat feeding in rodents. Our goal was to shed some light in a great deal of controversy regarding the whole-body consequences of non-nutritive sweeteners consumption, since the physiological mechanisms by which they impact energy balance and metabolic function still need to be clarified in animal models. To analyze the effects of the ingestion of a prototypical MNEI-sweetened beverage, designed for human consumption, i.e., with comparable sweetness to a fructose-based drink, we compared two groups of rats on the same high fat diet, which were administered drinking water sweetened with fructose (HFF) or MNEI (HFM), respectively. The HFF group consumed more sweetened water, also because fructose consumption is known to stimulate the sensation of thirst [23]. This behavior translated into a higher energy intake from beverages, but our results show that this induced a compensatory reduction of the calories introduced via high fat diet, as expected since it is known that the rat rodent model possesses a stringent control of the total energy intake. In fact, unlike humans who can introduce uncontrolled amounts of calories driven by physiological and/or emotional stimuli, rats alter their eating pattern in response to changes in the caloric density of food, by compensatory shift in the number and size of meals [24]. In line with these observations, when we calculated the total calorie intake for each group (chow + beverage), we found no significant variation between groups. This adaptive response could underlie the lack of significant differences in body energy compartments between HFF and HFM rats, for which body lipids, as well as epididymal fat, resulted unchanged. Additionally, the total body energy gain after 30 days of high fat feeding was the same between the two high fat groups, whatever the sweetener used in the beverage. 

The substitution of non-nutritive MNEI for caloric fructose in beverages, even if apparently irrelevant at whole body level, influenced glucose homeostasis. After a glucose load, both insulin and glucose plasma levels significantly increased in the HFF group, indicating that, even with hyperinsulinemia, the combined administration of fructose and high fat impairs glucose tolerance. By contrast, insulin response in HFM rats showed a trend very similar to the control LF rats, indicating that the consumption of MNEI, even in a high fat regimen, did not alter insulin sensitivity. The main significant differences among HFM and HFF groups, observed from the glucose tolerance tests, were the results showed between 20 and 150 min after glucose load. This suggest the onset of insulin resistance in HFF rats at the level of skeletal muscle since, following a meal, approximately one third of ingested glucose is taken up by the liver and the rest by peripheral tissues, primarily skeletal muscle via an insulin dependent mechanism [25]. It is well known that activation of the phosphoinositol 3-kinase/Akt pathway results in translocation of insulin-sensitive glucose transporter GLUT4 to the plasma membrane and in increase of glucose transport into muscle, and adipose tissue [26,27], maintaining glucose homeostasis. Reduced activation (via phosphorylation of Ser473) of Akt protein compared to controls was shown only in HFF rats, in agreement with data obtained from the glucose tolerance test. 

By considering the central role of TNF-α in obesity-induced insulin resistance in peripheral organs [26], we hypothesized an involvement of this cytokine in the onset of insulin resistance in HFF rats, but we found no variations of TNF-α levels between all the groups of rats in skeletal muscle and also in TNF-inducible transcription factor NFkB p65, a pathway normally antagonizing insulin signaling [28]. It has been also suggested that a lipotoxic profile could play a role in the pathogenesis of insulin resistance in this tissue [29]. We observed this kind of profile only in HFF rats. In detail, we showed an increase in total plasma cholesterol, as well as in triglycerides, only in HFF rats, compared to LF controls. This variation was completely absent in HFM rats, who showed levels of triglycerides significantly lower compared to the HFF group. The latter result was confirmed also at the skeletal muscle level, where significantly higher levels of triglycerides were detected only in HFF rats. A putative mechanism by which insulin resistance is induced in skeletal muscle involves toxic lipid metabolites, such as the sphingolipid ceramide, whose biosynthesis is increased by diets rich in saturated fatty acids [30]. Since skeletal muscle ceramides did not vary across the groups, regardless of the diet used, it is possible that the observed differences in insulin sensitivity between the two high fat groups are not explainable via this mechanism, at least in our experimental conditions. This hypothesis was supported by the fact that also the analysis of serine palmitoyl transferase protein content, which is responsible for the de novo ceramide production, did not show significant variations across experimental groups.

The results of this pilot study confirm the validity of the choice of Wistar rodent strain, despite the size of our statistical sample (*n* = 7) would need to be increased in further investigations on the effects of MNEI administration in high fat dietary regimen. The Wistar rat is usually a general multipurpose rat model and worked very well in the study, confirming our previous observation that this strain of rats shows a very low variability in response to different nutritional stimuli. In addition, the experimental protocol, as well as the housing of rats, simulates the condition of “physical inactivity” of the western lifestyle well, whose investigation was the target of this study.

It is well known that high fat diets modify the permeability of gut tissues to LPS [31], which in turn activate inflammation signaling in different organs. We observed a significant increase of plasma LPS only in HFF rats, while levels of plasma TNF-α were independent on the diet adopted. This result supports the data obtained from analysis of the colon, where the content of ZO-1, a tight junction protein of enterocytes, was lowered in HFF rats compared to LF controls. The reduction of ZO-1 content determines a defective intestinal barrier in HFF rats, leading to a higher translocation of LPS from the gut lumen into the circulatory system. In line with these findings, colon MPO activity, an index of local colonic inflammation, appeared significantly increased only in HFF rats. This hyper regulation of inflammation, known as metabolic endotoxemia [3], was only visible in HFF rats and not in rats drinking MNEI-sweetened water, which did not develop any metabolic impairment.

Further studies aimed at testing MNEI administration in low fat dietary regimen will be fundamental to upgrade the results of this pilot study, as well as an investigation of the potential effects of MNEI on gut microbiota composition.

## 5. Conclusions

In general, there is a lack of conclusive evidence-based research to discourage or to encourage the use of non-nutritive sweeteners on a regular basis. With this pilot study, we attempted to shed some light on the metabolic effects of the introduction of SPs into the diet. Even when maintaining a high fat diet regimen, we have observed that consumption of MNEI, instead of fructose in sweet beverages helped keep under control typical symptoms of diet-induced metabolic disorders. The most significant results were observed in terms of glucose tolerance, gut permeability and inflammation, on which MNEI exerted a rescue effect. Therefore, our preliminary results indicate that MNEI could represent a valid alternative to fructose, particularly when concomitant metabolic disorders such as diabetes and/or glucose intolerance are present, and may acquire more significance in humans, where the stringent control of total energy intake, typical of our rodent model, is absent. In this case, the higher energy intake coming from fructose in drinks is not compensated by a reduction in calorie assumption, leading to further exacerbation of the unhealthy consequences of unbalanced diet regimens. The promising results linked to MNEI consumption we observed in this pilot study support further investigations in light of their possible adoption for special diets regimens.

## Figures and Tables

**Table 1:** Composition of experimental diets and beverages.

	LF	HFF	HFM
DIET			
Standard chow *	50	50	50
Sunflower oil	2	2	2
Casein	9	14.8	14.8
AIN-76 mineral mix **	1.6	1.6	1.6
AIN-76 vitamin mix ***	0.4	0.4	0.4
Choline	0.1	0.1	0.1
Methionine	0.1	0.1	0.1
Cornstarch	36.8	10	10
Lard	---	21	21
Total weight, g	100	100	100
Water content, %	4	6	6
Metabolizable energy content, kJ ****	1398	1838	1838
Protein (J/100 J)	22.0	22.0	22.0
Lipids (J/100 J)	9.4	50.2	50.2
Carbohydrates (J/100 J)	68.6	27.8	27.8
BEVERAGES			
Water, ml	100	100	100
Fructose, g	---	10	---
MNEI, g	---	---	0.004

* Mucedola 4RF21, Italy, ** American Institute of Nutrition, 1977, *** American Institute of Nutrition, 1980. **** estimated by computation using values (kJ/g) for energy content as follows: protein 16.736, lipid 37.656 and carbohydrate 16.736. LF: low fat diet; HFF: high fat plus fructose diet; HFM: high fat plus MNEI diet.

**Table 2:** Energy balance in rats fed a low fat (LF), high fat plus fructose (HFF) or high fat plus MNEI (HFM) diet for 30 days.

	LF	HFF	HFM
Initial body weight, g	460 ± 23	461 ± 10	461 ± 12
Final body weight, g	499 ± 28	516 ± 10	518 ± 18
Body weight gain, g	39 ± 6	55 ± 4	57 ± 8
Epididymal fat weight, g (100 g_bw_)^−1^	1.5 ± 0.1	2.2 ± 0.2 *	2.1 ± 0.2 *
Body lipids, g (100 g_bw_)^−1^	14.7 ± 0.9	17.8 ± 0.9 *	17.7 ± 0.9 *
Water intake, mL	1324 ± 39	2018 ± 215 **	1371 ± 108 ^#^
Drink energy intake, kJ	0	2990 ± 363 *	0 *^#^
Diet energy intake, kJ	11,010 ± 293	8324 ± 371 **	10,750 ± 386 ^##^
Metabolizable energy intake, kJ	11,010 ± 293	11,314 ± 415	10,750 ± 386
Energy expenditure, kJ	9564 ± 251	10,072 ± 388	8960 ± 187
Energy gain, kJ	830 ± 100	1439 ± 140 *	1458 ± 143 *
Lipid gain, kJ	894 ± 88	1599 ± 120 *	1598 ± 150 *
Metabolic efficiency, %	7.3 ± 0.7	12.5 ± 2.0 *	13.8 ± 2.0 *

Table shows means ± SEM for 7 rodents in each group: low fat (LF), high fat plus fructose (HFF), high fat plus MNEI (HFM). * *p* < 0.05 compared to LF, ** *p* < 0.01 compared to LF, ^#^ *p* < 0.05 compared to HFF, ^##^ *p* < 0.01 compared to HFF (one-way ANOVA followed by Tukey post-test). bw: body weight.
